# Editorial: Metabolic signaling dysregulation and cognitive impairments in aging and Alzheimer's disease, volume II

**DOI:** 10.3389/fnagi.2023.1150101

**Published:** 2023-02-03

**Authors:** Tao Ma, Raymond Chuen-Chung Chang, Shannon L. Macauley

**Affiliations:** ^1^Department of Internal Medicine, Gerontology and Geriatric Medicine, Wake Forest University School of Medicine, Winston-Salem, NC, United States; ^2^Department of Physiology and Pharmacology, Wake Forest University School of Medicine, Winston-Salem, NC, United States; ^3^Department of Neurobiology and Anatomy, Wake Forest University School of Medicine, Winston-Salem, NC, United States; ^4^Laboratory of Neurodegenerative Diseases, LKS Faculty of Medicine, School of Biomedical Science, The University of Hong Kong, Pok Fu Lam, Hong Kong SAR, China

**Keywords:** Alzheimer's disease, aging, MCI, metabolism, biomarker, brain imaging, cognitive impairment

Alzheimer's disease (AD) is the most common dementia syndrome in the elderly and a devastating neurodegenerative disease without any cure available currently. AD patients usually present insidious onset and progressive cognitive impairments with unique post-mortem diagnostic brain pathology including accumulation of amyloid beta (Aβ) and hyper-phosphorylated tau proteins (Querfurth and LaFerla, 2010; Holtzman et al., 2011; Alzheimer's Association, 2022). Despite decades of research, the exact molecular/cellular mechanisms underlying AD pathogenesis remain elusive, which hampers development of effective therapeutic approaches and early diagnostic biomarkers for this devastating disease (Mullane and Williams, 2018; Alzheimer's Association, 2022; Miculas et al., 2022).

Regulation of energy metabolism homeostasis is an essential biological process that is critical for function and survival of all organisms. Metabolism involves numerous fuel sources and pathways that can be generally divided into two categories: the anabolic pathway which consumes energy and includes biosynthesis of molecules such proteins and lipids; and the catabolic pathway which releases energy and involves breakdown of complex molecules. The energy demands and metabolic rate of the brain are significant: with < 2% of the body weight, the brain consumes more than 20% of the body energy (Shulman et al., 2004; Hyder et al., 2013; Watts et al., 2018). Thus, the brain is uniquely susceptible to changes in energy availability and utilization, all of which put the brain at risk for damage or disease. Notably, dysregulation of energy metabolism and related signaling pathways in the nervous and other body systems are linked to synaptic failure and cognitive impairments in many aging-related CNS disorders including AD, Parkinson's disease, and mild cognitive impairment (MCI) (Lin and Beal, 2006; Arnold et al., 2018; Carroll and Macauley, 2019; Ryu et al., 2019; Wang et al., 2019; Wilkins and Swerdlow, 2021). Moreover, emerging evidence suggests that alterations in brain metabolism are not only related to neuronal injury but also changes in glial and vascular function associated with CNS disorders (Shippy and Ulland, 2020; Xiang et al., 2021; Ardanaz et al., 2022; Salvadó et al., 2022). Therefore, understanding the physiological and pathological roles of various metabolic processes and related signaling cascades in cognition may provide important insights into development of novel therapeutic targets and prognostic/diagnostic biomarkers for AD and related dementias (ADRDs).

The current Research Topic “*Metabolic Signaling Dysregulation and Cognitive Impairments in Aging and Alzheimer's Disease, Volume II*” serves as the 2nd part of the article collection series (under the same title) and highlights both basic and clinical studies in the relevant field. In total, seven original research articles from investigator groups across the world using multiple state-of-art scientific approaches reported AD- or ADRD-related metabolic signaling alterations in rodent models or human subjects.

The Qi Guo group (Zhao et al.) analyzed plasma metabolites profile in the MCI patients by using a high-resolution mass spectrometry metabolomics approach. The results indicate an association between dysregulated sphingolipid metabolism and behavioral performance in the MCI patients, providing insights into development of novel blood biomarker for MCI.

The Lior Greenbaum group (Manzali et al.) examined the relationship between AD polygenic risk score (PRS) and longitudinal decline of cognition function in older adults with type 2 diabetes (T2D). Interestingly and contrary to the authors' hypothesis, the analysis revealed no significant association between the AD PRS and multiple measurements in older adults with T2D such as cognitive decline, hippocampal volume, and Aβ burden. Their findings indicate a complex relationship between AD genetic susceptibility and cognitive impairments in T2D.

The laboratory of Tao Ma (Kasica et al.) reported that global knockout of the mRNA translation factor eukaryotic elongation factor 2 (eEF2) kinase (eEF2K) led to improvement of cognitive function in a mouse model of AD without affecting the brain Aβ pathology. The study provides further evidence to suggest that targeting eEF2K signaling dysregulation might be a feasible therapeutic approach for ADRDs.

Kotkowski et al. investigated, in a cohort of Mexican American adults, potential association of metabolic syndrome (MetS) biometric components and the gray matter volume (GMW) in different brain regions. They found that decreased GMV across all the examined brain regions in the MetS patients is correlated best with waist circumference. Surprisingly, posterior cerebellum is the brain region mostly correlated to the MetS biometric components.

Weise et al. analyzed the data of the MCI participants from the AD Neuroimaging Initiative (ADNI) study and reported a correlation of higher body mass index (BMI) with greater regional cerebral metabolic rate for glucose (rCMGgl) and slower cognitive decline in MCI. Such association is not related to leptin levels. Future studies are necessary to illustrate the mechanisms underlying such correlations between brain glucose utilization and body weight.

The laboratory of Jungsu Kim (Smith et al.) reported their unexpected findings revealing novel roles of the AD risk gent Abelson interactor family member 3 (Abi3) in metabolism. They found that the Abi3 knockout mice exhibited impaired energy expenditure, increased body weight and body fat, and impaired glucose tolerance and insulin sensitivity. The study may contribute to our understanding of the metabolic factors in AD development.

Kurano et al. examined postmortem human brains to understand potential association of the bioactive lipid regulation with the AD pathology. The study showed distinct pattern of bioactive lipid modulation correlated to AD pathogenesis.

In summary, accumulating evidence indicates a link between metabolic signaling dysregulation and cognitive impairments associated with aging and Alzheimer's disease. An important goal of this Research Topic in *Frontiers in Aging Neuroscience* is to increase overall interest in this under- investigated area of research and bring investigators from both basic science and clinical research fields. We are encouraged and excited by the findings reported in these articles and look forward to future in-depth studies on the topic to facilitate our underlying of the mechanisms underlying aging and ADRDs.

## Author contributions

TM wrote the manuscript. RC and SM edited the manuscript. All authors contributed to the article and approved the submitted version.
